# LATEST ADVANCES IN UNDERSTANDING THE RELATIONSHIPS BETWEEN FRAILTY AND COGNITIVE IMPAIRMENT

**DOI:** 10.1093/geroni/igz038.1463

**Published:** 2019-11-08

**Authors:** Qianli Xue, Michelle C Carlson

**Affiliations:** 1 Department of Medicine, School of Medicine, Johns Hopkins University, Baltimore, Maryland, United States; 2 Johns Hopkins Bloomberg School of Public Health, Baltimore, Maryland, United States

## Abstract

As two of the most common geriatric conditions, frailty and cognitive impairment often coexist, and are known to predict poor health outcomes separately and jointly. The link between frailty and cognitive impairment may result from the fact that many of the aging processes underlying frailty may also be responsible for brain aging and cognitive decline. What is unknown is: (1) whether frailty as a measure of physiological resilience is predictive of dementia above and beyond neuropathology and cognitive decline; (2) whether there are individual characteristics that uniquely identify separate vs. joint presence of physical frailty and cognitive impairment. To begin to address these questions, Talk 1 reviews concept of frailty in relationship to reserve and resilience and discusses theoretical underpinnings of three integrated phenotypes of physical and cognitive impairment; Talk 2 uses data from two epidemiological cohorts to study the relationship between frailty and dementia after accounting for neuropathology. Talk 3 uses data from the Gait & Brain Study to compare the strength of associations of cognitive impairment alone vs. cognitive impairment plus physical frailty (or gait performance) with incident dementia. Talk 4 uses data from the National Health and Aging Trends Study to develop a U.S. national profile on the intersection between physical frailty and cognitive impairment in community-dwelling older adults. Together, findings from this study help elucidate the interconnection between physical frailty and cognitive impairment, as well as clinical utility of joint consideration of cognitive decline and physical frailty for predicting risk of dementia.

